# Multi-Level Determinants of Parasitic Fly Infection in Forest Passerines

**DOI:** 10.1371/journal.pone.0067104

**Published:** 2013-07-10

**Authors:** Darío Ezequiel Manzoli, Leandro Raúl Antoniazzi, María José Saravia, Leonardo Silvestri, David Rorhmann, Pablo Martín Beldomenico

**Affiliations:** Laboratorio de Ecología de Enfermedades, Instituto de Ciencias Veterinarias del Litoral, Universidad Nacional del Litoral – Consejo de Investigaciones Científicas y Técnicas (UNL – CONICET), Esperanza, Santa Fe, Argentina; Universidade Federal de Minas Gerais, Brazil

## Abstract

The study of myiasis is important because they may cause problems to the livestock industry, public health, or wildlife conservation. The ecology of parasitic dipterans that cause myiasis is singular, as they actively seek their hosts over relatively long distances. However, studies that address the determinants of myiasis dynamics are very scarce. The genus *Philornis* include species that may be excellent models to study myiasis ecology, as they exclusively parasitize bird nestlings, which stay in their nests until they are fully fledged, and larvae remain at the point of entry until the parasitic stage is over, thus allowing the collection of sequential individual-level infection data from virtually all the hosts present at a particular area. Here we offer a stratified multi-level analysis of longitudinal data of *Philornis torquans* parasitism in replicated forest bird communities of central Argentina. Using Generalized Linear Models and Generalized Linear Mixed Models and an information theory approach for model selection, we conducted four groups of analyses, each with a different study unit, the individual, the brood, the community at a given week, and the community at a given year. The response variable was larval abundance per nestling or mean abundance per nestling. At each level, models included the variables of interest of that particular level, and also potential confounders and effect modifiers of higher levels. We found associations of large magnitude at all levels, but only few variables truly governed the dynamics of this parasite. At the individual level, the infection was determined by the species and the age of the host. The main driver of parasite abundance at the microhabitat level was the average height of the forest, and at the community level, the density of hosts and prior rainfall. This multi-level approach contributed to a better understanding of the ecology of myiasis.

## Introduction

A vast variety of dipterans affects the health of animals, including humans, either by acting as disease vectors [1], [2] or through parasitism [3]. Some flies with parasitic immature stages cause myiasis, a tegument or cavity infection with larvae, which may represent great economic losses for livestock industry (e.g. species of *Lucilia* [4]), public health concern (e.g. *Dermatobia hominis* [3]), or contribute to wildlife species declines (e.g. *Philornis downsi* [5]).

Parasites that cause myiasis are among those that actively seek their hosts over relatively long distances, which results in a particular life-cycle and natural history. Despite this, the ecology of myiasis has seldom been studied in detail. Some research has been conducted, but each study only sheds light on few selected factors that influence myiasis occurrence. Current data available include associations of parasitic dipterans with climatic factors [6], [7], host density [7], [8], and nest and host characteristics [8]–[11]. Myiasis ecology studies that jointly encompass the analysis of several factors at different levels of biological hierarchy would provide us with a better understanding of the web of causation behind myiasis dynamics, describing the relative contribution of each variable while enabling the assessment of interactions between factors at different levels.

*Philornis* Meinert (Diptera: Muscidae) is a genus of flies that includes several parasitic species, whose larvae parasitize New World bird nestlings [12]. Most parasitic *Philornis* spp. cause subcutaneous myiasis, with burrowing larvae that feed on nestling blood, tissue and fluids [13]. These parasites harm nestlings, causing mortality and reduced fitness and growth [7], [10], [14]. Subcutaneous *Philornis* larvae constitute a very interesting model to study the ecology of myiasis, as they principally parasitize bird nestlings, which stay in their nests until they are fully fledged, and larvae are easily spotted and remain at the point of entry until the parasitic stage is over, thus allowing the collection of sequential individual-level infection data from virtually all the hosts present at a particular area.

Here we offer a stratified multi-level analysis of longitudinal data of *Philornis torquans* (Nielsen, 1913) parasitism in two forest bird communities of central Argentina. The effect of host-, microhabitat- and community-level factors and some of their relevant interactions were assessed. Although many host and environmental variables might be associated with the occurrence of this parasite, it is expected that the integrative approach employed will enable us to pinpoint the major drivers of infection risk. While studies that place the focus on a limited group of risk factors are appropriate to demonstrate causal associations between a putative cause and a parasite, only integrative approaches allow us to assess what variables explain most of the variance when all of them are interacting together. We predict that, although many host and environmental factors will be found to be associated with *Philornis* infection, only a few truly drive the dynamics of parasitism.

## Materials and Methods

### Study area

The data were collected from two native forest patches located in the center of Santa Fe Province (Argentina). One is a reserve belonging to Universidad Nacional del Litoral (centre at 60° 55′ W, 31° 23′ S), the other one, a private field known as “Mihura” (centre at 60° 47′ W, 31° 30′ S) (Mr. Kling and Mr. Gimenez kindly allowed us to carry out our fieldwork in their property). The distance between the two sites is 20km. The area sampled within each site was 40 ha. Both sites represent relicts of the biogeographic province ‘El Espinal’ and are located alongside the Salado River. The climate in the region is Pampean Temperate, with an average annual temperature of 18°C (mean minimum  = 12°C; mean maximum  = 23°C) (extracted from www.climayagua.inta.gov.ar). These forests are breeding grounds for around 100 bird species, mostly Passeriformes [15]. More than 20 species have been found to be parasitized by *Philornis torquans* larvae in this region, but only three account for over 90% of the records [7].

### Data collection

The fieldwork to obtain the data spanned the breeding seasons of 2008–2009 (September – April) and 2009–2010 (September – May). As previous observations showed that the parasitic larvae do not occur until around 10 weeks after the first eggs of the season are laid, the observations from the first weeks (September and October) were not used in the analysis. Every week, each area was exhaustively examined for active nests. All broods detected were thoroughly examined on a weekly basis. Nestlings from species known to be the predominant hosts in the region (i.e. *Pitangus sulphuratus* L., *Phacellodomus ruber* Vieillot, *Phacellodomus sibilatrix* Sclater) [7], were sampled three times a week (every 2–3 days), for a more detailed collection of information on individual level variables. All nestlings were thoroughly examined to determine the presence of *Philornis* larvae, which were recorded and classified into developmental stages from instar one to three. In the two breeding seasons (totaling 70 weeks) and from both sites, we collected 6629 observations from 3255 nestlings (1400 broods) of 57 bird species. Of them, 2291 observations belonged to predominant hosts (43 broods of *Ph. ruber*, 79 broods of *Ph. sibilatrix* and 70 broods of *Pi. sulphuratus*). Other species that contributed with considerable amount of data (1423 observations) were *Furnarius rufus* Gmelin (85 broods), *Paroaria coronata* Miller (89 broods) and *Sicalis flaveola* L. (105 broods). The data will be made freely available upon request.

The factors evaluated were chosen for their biological sense on the basis of current knowledge on the bio-ecology of birds, dipterans and ectoparasites. A preliminary exploratory analysis allowed us to identify independent variables that were highly correlated between them, of which we chose the most strongly associated with the response to avoid co-linearity problems.

The data obtained at the individual level were species, age (days from hatching), body mass (g), tarsus length (mm) and body mass index (body mass/tarsus length). Additionally, once a week, a small blood sample (<10 µl) was taken from a nail cut into a heparinized capillary tube to carry out haematological analyses as described by Lucas and Jamroz [16].

Microhabitat was referred to as the environment surrounding the brood, including the more immediate (e.g. nest material) and less proximal (e.g. vegetation structure) factors. Nest variables were the species which built the nest, nest external material (grass, mud, sticks, vegetable fiber or wood), chamber material (feathers, grass, sticks, vegetable fiber or wood), degree of environmental exposure of the chamber (open/closed), brood size, nest height (meters above the ground), and nest support (tree species, ground, etc.). The immediate surroundings of the nest were classified according to proximity to water bodies and the vegetation structure, which consisted of determining presence/absence and average height of three different vegetation strata (trees, bushes and grass), and their type (dominant species for each vegetation strata). To this aim, the study sites were characterized *in situ*, measuring the proximity to water bodies and assessing the vegetation structure for reference nests, so that all the area of each site was covered. Nests that were in close proximity to a reference nest (<30 m) received the same environmental values.

The variables recorded at the community level were weekly precipitation (mm; from the closest meteorological station), weekly mean temperature and relative humidity (using HOBO Pro v2 data loggers placed in the middle of each study area), and total and specific bird nestling density (number of nestlings per Ha in total and by group of bird species).

Finally, from one of the sites (the reserve), we have systematically recorded data on brood density and *Philornis* parasitism from the breeding season of 2006–2007 through 2011–2012 (6 years), which enables a preliminary analysis to explore inter-annual fluctuation trends in parasitism and their associations with bird community structure and climatic variables, giving a different time-scale context to the results.

For a detailed description of all variables see Table S1.

### Statistical analysis

The data analyses were conducted at three hierarchical levels, the individual, the microhabitat (nest and surroundings), and the community. Instead of approaching the analysis using a single hierarchical model, which is the usual method used for multi-level analyses [17], we utilized a stratified analytical approach, examining different study units separately, as we judged it more informative and robust. The analyses at lower levels placed the focus on variables acting at that level (e.g. host age), but conveniently included variables from higher levels to avoid potential confounding phenomena (e.g. if more rainfall increases both *Philornis* intensity and nestling body growth, ignoring rainfall in the data analysis at the individual level will produce a distorted association between tarsus length and parasitism). Relevant interaction terms were also assessed.

The analyses were conducted using generalized linear mixed models (GLMM) and generalized linear models (GLM) with negative binomial responses. The software used was R (The R Project for Statistical Computing; http://www.r-project.org) and the specific package was glmmADMB (function ‘glmmadmb’).

Model selection was carried out using an information theory approach, following Burnham & Anderson [18]. Starting from a global (maximum) model, model selection and comparison was carried out in a stepwise manner using Akaike information criteria (AIC) or, when overdispersion was present, Quasi-Akaike information criteria (QAIC) [19]. Our approach to model selection was as follows: firstly, all models with AIC values no greater than 5 units compared to the best model were considered. Then the ΔAICi between models was calculated as:
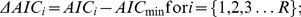
where *AIC_min_* denotes the AIC value of the best model [20]. *ΔAIC_i_* was used to estimate the relative likelihood (*l_i_*)



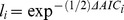



The latter allowed the calculation of the weight (*w_i_*) of each model
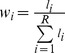



Finally, the models were ordered according to their *w_i_*, and the top models that summed 0.9 (cumulative *w_i_*) were selected [18]. The multimodal inference was done using the weighed mean of the *β* coefficient and its standard error. The terms that were considered significant were those with a coeficient's 95% confidence interval that did not include 0.

The analysis at the individual level was done with *GLMM* and included only the data collected from predominant hosts (*Pi. sulphuratus*, *Ph. ruber*, *Ph. sibilatrix*). The study unit was the individual chick at a given time (*t_i_*). The response variable was larval abundance per nestling [21], i.e. the number of larvae that were parasitizing the chick at *t_i_* (the value for a non-parasitized host was ‘0’). The independent variables of interest were host species, age with linear and quadratic terms, previous measures of condition (taken at previous visits: 2–3 days previously, *t_−1_*; or 4–5 days previously, *t_−2_*), which included body mass, body mass index, and total red blood cells and white blood cells. The potential confounders and interactions included are described in Table S2. Briefly, they were nest variables such as brood size and nest material, week and its quadratic term to account for differences along the breeding season, and the community variables site, year, minimum and maximum weekly mean temperatures, and weekly cumulative precipitation, each at different time lags (*t_0_* through *t_−6_*). We also controlled for the density of all or each of the three predominant host species at different time lags (*t_0_* through *t_−4_*). In addition, densities of nestlings of unsuitable host species (non-passerines) and an index of biodiversity (Shannon's) [22] were assessed at different time lags, to explore a possible ‘dilution effect’ [23]. For variables that included time lags, the mean or sum estimates for a week were calculated using the values from seven consecutive days. For lags at *t_0_*, the values included were those of the day of the observation (*day_0_*) plus the ones of the preceding 6 days (*day_−1_*, *day_−2_*, *day_−3_*, *day_−4_*, *day_−5_*, and *day_−6_*); for lags at *t_−1_* we used *day_−7_* through *day_−13_*, and so on. Relevant two-way interactions (between two variables of interest or a variable of interest and a potential confounder) were assessed (see Table S2). To account for the lack of independence between observations from the same nest and nestling we included the nested random effect ‘nest id/individual id’.

At the microhabitat level, the analysis was also done with *GLMM* and included data collected from the six most abundant passerine species (the predominant ones plus *F. rufus*, *P. coronata* and *S. flaveola*). The study unit was a single brood at *t_i_.* The response variable was the mean larval abundance per bird, i.e. the number of larvae/number of nestlings in the brood. The independent variables of interest were the brood species, the species that built the nest, brood size, presence of nestlings of other species (i.e. parasitic: cowbirds, *Molothrus bonaerensis* Gmelin and striped cuckoos, *Tapera naevia* L.), environmental exposure of the nest chamber (open/closed), nest height, external and internal nest material (see above), and presence/absence, type and average height of different vegetation strata (trees, bush and grass). Quadratic terms were added to account for non-linearity of continuous independent variables (e.g. nest height). The potential confounders included were the same community level variables assessed for the individual level models (see Table S2). Also, relevant two-way interactions were assessed. The random effect included was ‘nest id’.

A *GLM* was used to analyze the data at the community level. In this case, the study unit was the whole bird community (either from the reserve or Mihura) at a given week (*t_i_*). The response variable was the mean larval abundance per bird (total larvae *t_i_*/number of nestlings *t_i_*) ×10 (i.e. the mean number of larvae per ten nestlings at a given week). The independent variables of interest were week, minimum and maximum weekly mean temperatures and humidity at different time lags (*t_0_* through *t_−6_*), weekly cumulative precipitation (sum of mm fallen at *t_i_*) at the same time lags, and density of hosts at different time lags (*t_0_* through *t_−6_*), as follows: potential hosts (22 spp.; all host species that were found to be parasitized by *P. torquans*), predominant hosts (the 3 spp. named above), unsuitable hosts (non-passerines), and single species: *Pi. sulphuratus*, *Ph. ruber* and *Ph. sibilatrix*. Past abundance per bird of third instar larvae (*t_−1_* through *t_−6_*) was also assessed. The corresponding quadratic terms were also included. The potential confounders assessed were site and year. The relevant two-way interactions included were those between year, site, temperature, precipitation and host densities at different time lags (see Table S2).

For inter-annual comparisons we used data from seasons 2006–2007 through 2011–2012. Each season, the sampling started the week of September 21^st^, and ended when no more breeding was detected, except in 2006–2007, when the fieldwork was interrupted at the beginning of March because the study area was flooded, and in 2010–2011, when it had to be interrupted by the end of January due to health problems of the field workers. To account for this we used data from October to February. The analysis consisted in Spearman's Rho correlation tests between annual measures of parasitism (annual prevalence and mean parasite abundance per nestling) and climatic and host abundance data.

### Ethics Statement

The animal care and treatments used in this study were approved by the Bioethical Committee of the School of Veterinary Medicine of Universidad Nacional del Litoral (PROTOCOL No. 02/08).

## Results

Larvae of *P. torquans* were found in 22 out of 57 species examined. At the individual level, the highest levels of parasitism were observed in *Pi. sulphuratus*, with 41.2% parasitized and a mean infection intensity of 11.1 larvae/nestling, followed by *Ph. ruber* with a prevalence of 12.6% and a mean intensity of 13.4 and *Ph. sibilatrix*, with 10.6% prevalence and a mean intensity of 3.6. At the brood level, the prevalence and mean intensity were, respectively, 44.9% and 45.0 larvae/brood for *Pi. sulphuratus*; 35.3% and 8.9 for *Ph. sibilatrix;* 24.2% and 53.8 for *Ph. ruber*; 24.2% and 15.2 for *F. rufus*; 16.1% and 8.7 for *Pa. coronata* and 10.7% and 3.2 for *S. flaveola*. For a summary of the mean parasite abundance and prevalence by year, see Table 1.

**Table 1:** Annual estimates of *P. torquans* parasitism, host abundance and climatic variables observed at one of the study sites (the reserve) from 2006 through 2012 during the first two thirds of each breeding season.

Season	Prevalence	MLA^a^	CP^b^	MMinT^c^	MMaxT^d^	*Pi. sulph* A^e^	PHA^f^
2006–2007	0.314	4.24	921	20.04	29.60	28	47
2007–2008	0.264	2.23	436	19.91	29.65	45	108
2008–2009	0.264	1.79	372	19.70	30.26	25	73
2009–2010	0.333	3.97	920	19.18	28.94	50	100
2010–2011	0.205	1.83	475	18.41	29.98	7	64
2011–2012	0.229	2.24	581	19.19	29.73	16	41

a Mean larvae abundance.

b Cumulative precipitation (mm).

c Mean Minimum temperature (C°).

d Mean Maximum temperature (C°).

e Pitangus sulphuratus abundance.

f Predominant host abundance.

### Individual-level variables associated with infection

The individual variables that consistently remained as important predictors of *P. torquans* infection were host species, age (linear and quadratic terms) and the interaction between them (Table 2).

**Table 2:** Parameters of the best model describing individual factors associated with *P. torquans* infection in its predominant hosts.

Term	Coefficient	Standard error	95%CI LB* ^1^	95%CI UB* ^2^
**Intercept**	**−10.205**	**2.300**	**−14.714**	**−5.696**
**Host sp** (***Ph. ruber***)**^a^**	**4.470**	**1.456**	**1.616**	**7.323**
**Host sp** (***Ph. sibilatrix***)**^a^**	**1.906**	**0.867**	**0.206**	**3.606**
**Age**	**0.569**	**0.148**	**0.280**	**0.858**
**Age^2^**	**−0.024**	**0.007**	**−0.038**	**−0.010**
**Host sp** (***Ph. ruber***)* **age**	**−0.281**	**0.106**	**−0.489**	**−0.072**
**Host sp** (***Ph. sibilatrix***)* **age**	**−0.468**	**0.087**	**−0.639**	**−0.296**
**Host sp** (***Ph. ruber***)* ***Pi. sulphuratus*** ** dens _t−2_**	**−20.967**	**5.949**	**−32.627**	**−9.307**
**Host sp** (***Ph. sibilatrix***)* ***Pi. sulphuratus*** ** dens_t−2_**	**−33.110**	**9.432**	**−51.598**	**−14.622**
*Pi. sulphuratus* dens _t0_	8.962	2.154	4.741	13.184
*Pi. sulphuratus* dens _t−2_	0.655	1.892	−3.054	4.364
*Ph. ruber* dens _t−2_	−19.578	3.659	−26.749	−12.407
Max. Temp _t−3_	0.200	0.073	0.058	0.342
Rain _t−5_	0.007	0.003	0.000	0.013
Site (reserve)^b^	1.961	0.675	0.639	3.284

* ^1^: lower bound; *^2^: upper bound.

a: compared to *Pitangus sulphuratus* (reference host); b: compared to Mihura (reference site).

Terms in bold indicate the variables of interest. Significant coefficients are underlined.

Host sp: predominant host species; Age: nestling age in days; *Ph. ruber* and *Pi. sulphuratus* dens: *Phacellodomus ruber* and *Pitangus sulphuratus* nestling density, respectively; Max.temp: weekly mean maximum temperature; Rain: weekly sum of precipitation; t0 – t−5 refer to time lags (0 =  current week; 5 = 5 weeks previously); site: studies sites.

**Figure 1:**
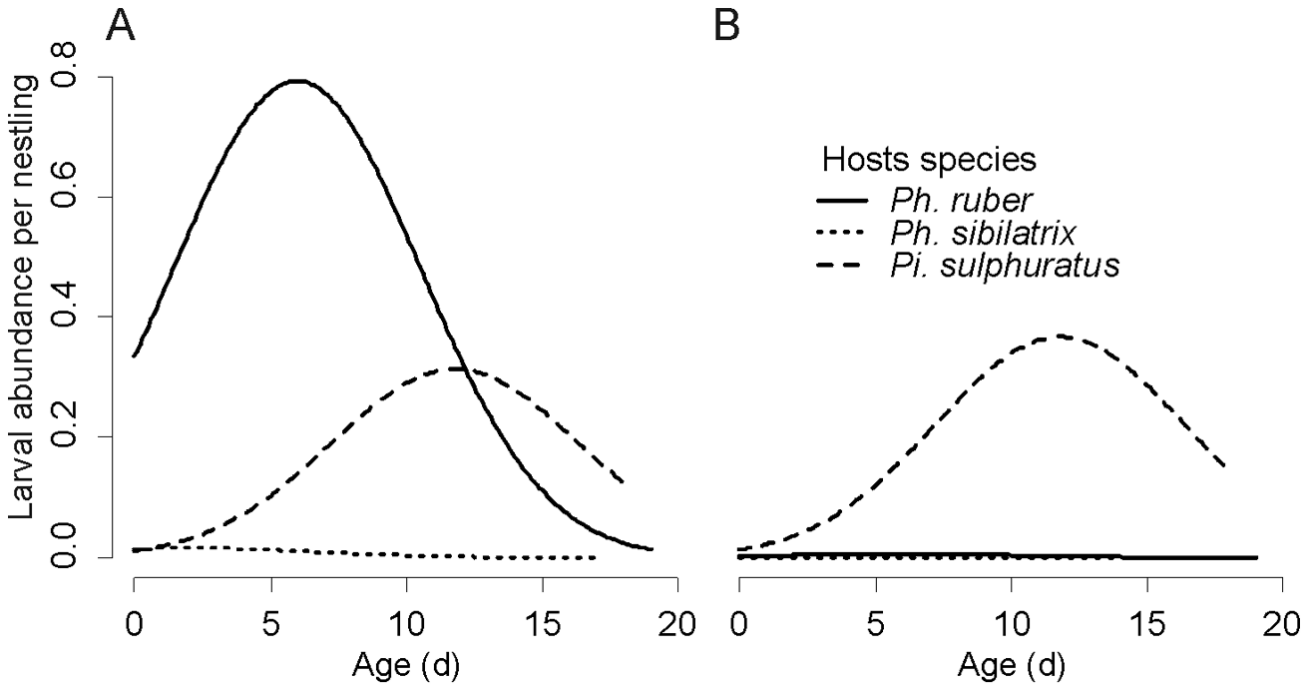
*Philornis* abundance as predicted by the best individual-level models. The figure depicts the interaction between host species and age and host species and *Pi. sulphuratus* density 2 weeks previously. A) scenario predicted for a low preceding *Pi. sulphuratus* density (*Pi. sulphuratus* density _t−2_ was set at 0.03 nestlings per Ha); B) scenario predicted for a high preceding *Pi. sulphuratus* density (0.3 nestlings per Ha).

The pattern of infection varied with the age of the nestling, but it was different in each of the three species. The larval abundance per nestling increased immediately after hatching to reach a peak and decrease afterwards, but the peak occurred at different times for each host species: at 2 days of age for *Ph. sibilatrix*, 6 for *Ph. ruber* and 12 for *Pi. sulphuratus* (Figure 1).

There was a strong interaction between species and *Pi. sulphuratus* density two weeks previously. While the larval abundance per nestling in *Ph. sibililatrix* was consistently lower than in the other two species, *P. torquans* parasitized *Ph. ruber* mainly two weeks after the densities of *Pi. sulphuratus* had been low (Figure 1), indicating there is plasticity in host preference dependent on host availability.

All other individual variables assessed were not significantly associated with *Philornis* larva abundance.

### Microhabitat-level variables associated with infection

**Figure 2:**
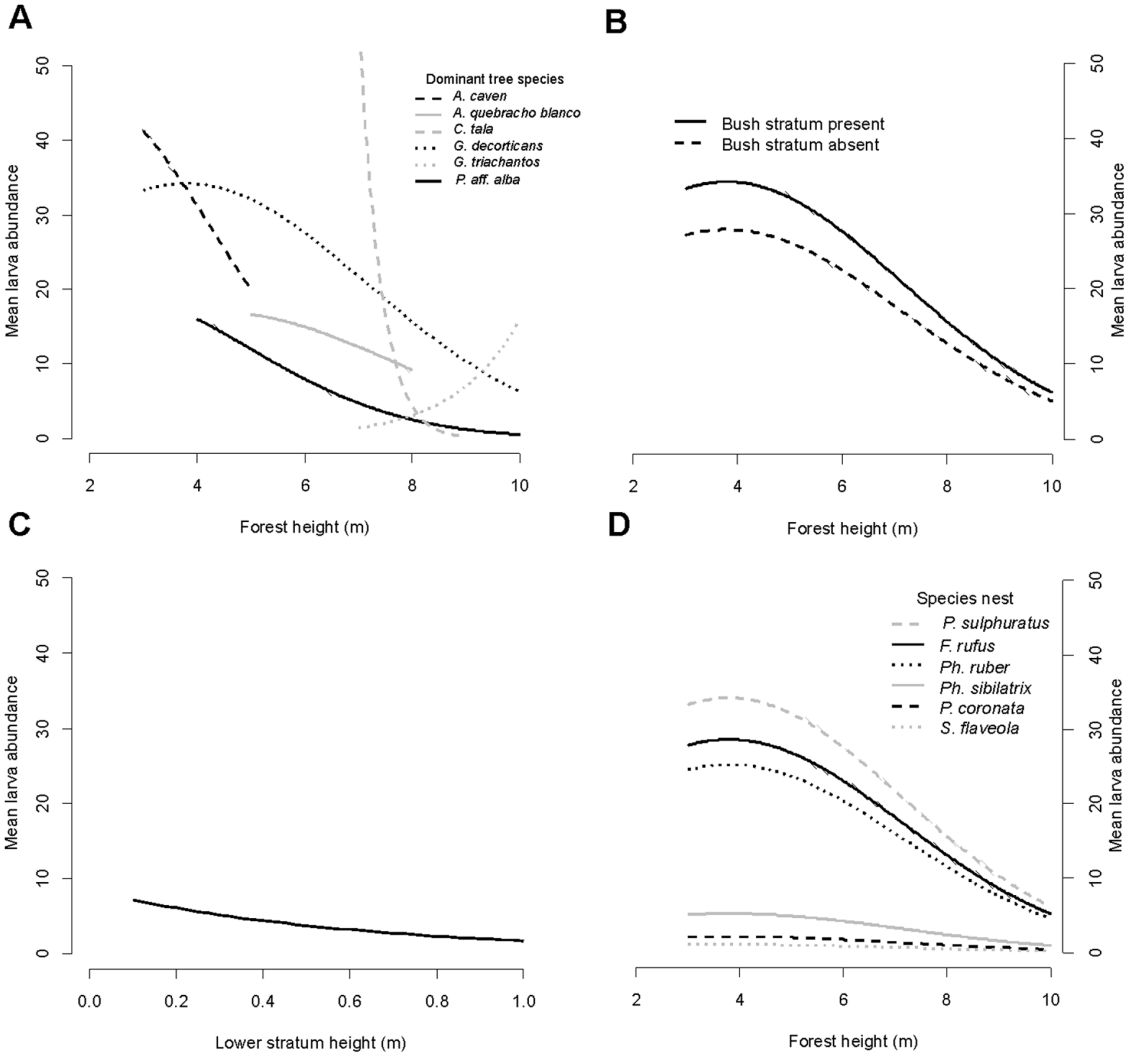
Mean *Philornis* abundance as predicted by the best microhabitat-level models. A) Relationship between average tree height and mean parasite abundance by dominant tree species. Prediction for *Pi. sulphuratus* broods. Each line spans the range of forest heights recorded for a particular dominant tree species; B) Effect of the presence of medium vegetation stratum (bush) on the mean parasite abundance. Prediction for *Pi. sulphuratus* broods in forests dominated by *G. decorticans*; C) Effect of the low vegetation stratum (grass) height. Prediction for *Pi. sulphuratus* broods in forests dominated by *G. decorticans*; D) Effect of the brood species on the mean parasite abundance. Prediction for forests dominated by *G. decorticans*. In all predictions, average values were used for continuous variables in the model.

The analysis at this level was conducted using 643 observations from 312 broods of the six most abundant species. The models with best goodness of fit are shown in Table S3. Broods of *Pi. sulphuratus*, *F. rufus* and *Ph. ruber* were more parasitized than those of *Pa. coronata, Ph. sibilatrix* and *S. flaveola* (Table 3; Figure 2).

**Table 3:** Variables of interest of the best models describing microhabitat (nest and surrounding) factors associated with mean *P. torquans* abundance in a brood.

Term	Coefficient	Standard error	95%CI LB* ^1^	95%CI UB* ^2^
Intercept	−8.128	3.667	−15.315	−0.940
**Brood sp** (***F. rufus***)^a^	**−0.179**	**0.415**	**−0.991**	**0.634**
**Brood sp** (***Pa. coronata***)^a^	**−2.758**	**0.373**	**−3.488**	**−2.028**
**Brood sp** (***Ph. ruber***)^a^	**−0.304**	**0.376**	**−1.041**	**0.433**
**Brood sp** (***Ph. sibilatrix***)^a^	**−1.878**	**0.340**	**−2.544**	**−1.213**
**Brood sp** (***S. flaveola***)^a^	**−3.466**	**0.343**	**−4.139**	**−2.793**
**O/c nest** (**open**)**^b^**	**0.025**	**0.026**	**−0.026**	**0.075**
**Tree sp** (***P. aff. alba***)**^c^**	**8.017**	**3.307**	**1.536**	**14.498**
**Tree sp** (***A. caven***)**^c^**	**8.744**	**4.627**	**−0.325**	**17.813**
**Tree sp** (***G. decorticans***)**^c^**	**8.109**	**3.215**	**1.807**	**14.411**
**Treesp** (***A. quebracho-blanco***)**^c^**	**7.168**	**4.209**	**−1.082**	**15.418**
**Tree sp** (***C. tala***)**^c^**	**13.826**	**55.645**	**−95.240**	**122.891**
**Tree height**	**0.773**	**0.454**	**−0.118**	**1.664**
**Tree height^2^**	**0.003**	**0.021**	**−0.039**	**0.045**
**Tree sp** (***P. aff. alba***)* **tree height**	**−0.552**	**0.201**	**−0.945**	**−0.159**
**Tree sp** (***A. caven***)* **tree height**	**−0.456**	**0.458**	**−1.353**	**0.441**
**Tree sp** (***G. decorticans***)* **tree height**	**−0.438**	**0.192**	**−0.813**	**−0.062**
**Tree sp** (***A. quebracho-blanco***)* **tree height**	**−0.372**	**0.312**	**−0.984**	**0.240**
**Tree sp** (***C. tala***)* **tree height**	**0.250**	**0.753**	**−1.227**	**1.726**
**Tree sp** (***P.aff.alba***)*(**tree height**)**^2^**	**−0.060**	**0.018**	**−0.094**	**−0.025**
**Tree sp** (***A. caven***)*(**tree height**)**^2^**	**−0.088**	**0.070**	**−0.224**	**0.049**
**Tree sp** (***G. decorticans***)*(**tree height**)**^2^**	**−0.047**	**0.017**	**−0.079**	**−0.014**
**Tree sp** (***A. qubracho-blanco***)*(**tree height**)**^2^**	**−0.049**	**0.035**	**−0.117**	**0.019**
**Tree sp** (***C. tala***)*(**tree height**)**^2^**	**−0.243**	**0.773**	**−1.757**	**1.272**
**p/a bush** (**presence**)**^d^**	**0.2053**	**0.102**	**0.006**	**0.404**
**p/a grass** (**presence**)**^e^**	**0.503**	**0.356**	**−0.195**	**1.200**
**Grass height**	**−1.606**	**0.551**	**−2.686**	**−0.525**
**Parasitic bird** (**presence**)**^f^**	**0.026**	**0.081**	**−0.133**	**0.185**

(Variables included in the model for adjustment are shown in Table S5).

* ^1^: lower bound; *^2^: upper bound.

a : Compared to *Pi. sulphuratus* species (brood sp reference); ^b^: Compared to nest surrounded with vegetation (reference);^c^: *Gledittsia triacanthos* (tree sp reference);^d^: Compared to bush absence (reference level); ^e^: Compared to grass absence (reference level); ^f^: Compared to parasitic bird absence (reference level).

Terms in bold indicate the variables of interest. Significant coefficients are underlined.

Reference: brood sp: Brood species parasitized by *P. torquans*; o/c nest: nest surrounded or not by vegetation; tree sp: dominant tree species in the community; tree height: mean height of dominant tree; p/a bush: presence or absence of medium stratum; p/a grass: presence or absence lower stratum; grass height: mean height of lower stratum; parasitic bird: presence or absence of parasitic bird.

Regarding the vegetation surrounding the nests, there was a significant interaction between dominant tree species and forest height (with both linear and quadratic terms), indicating that the occurrence of *P.torquans* declined with the height of the forest, except in those dominated by *Gledittsia triacanthos* L., the only exotic tree species (Table 3; Figure 2). In low to medium height forests, the mean larval abundance was greater when the dominating species were *Acacia caven* Molina, *Geoffrea decorticans* (Gill. ex Hook. & Arn.) Burkart and *Celtis tala* Gillies ex Planch than in forests of *Prosopis aff. alba*, *Aspidosperma quebracho-blanco* Schltr. and *G. triacanthos.* Nests surrounded by bush had significantly more larvae than those built in the absence of this vegetation cover, while the association with the herbaceous stratum height was negative (Table 3; Figure 2). The relationship between the microhabitat variables and *Philornis* did not depend on the brood species (the relevant interaction terms were not significant).

### Community-level variables associated with infection

**Figure 3:**
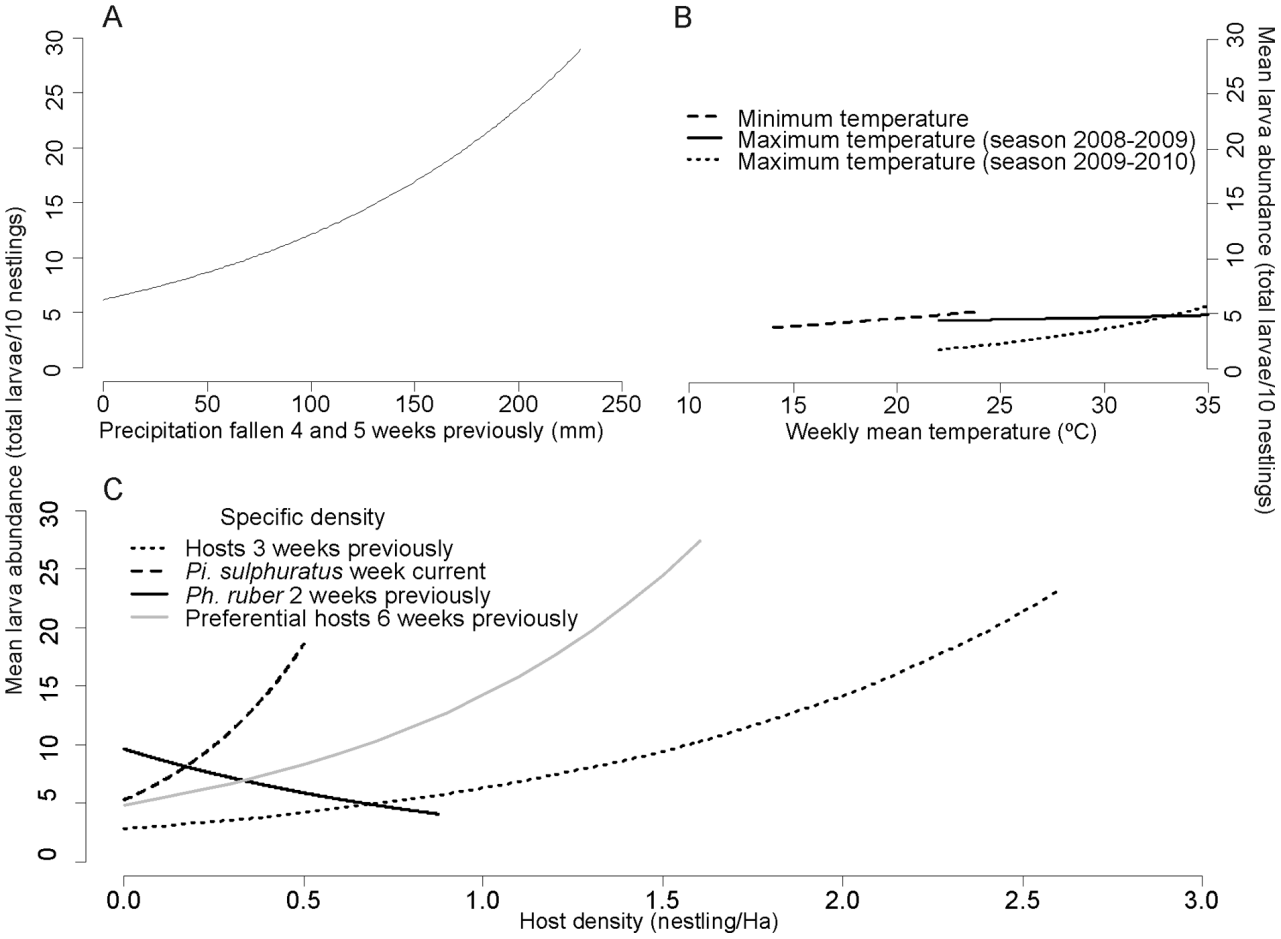
Mean *Philornis* abundance as predicted by the best community-level models. A) Effect of precipitation (mm) fallen 4 and 5 weeks previously. B) Effect of weekly mean temperature. C) Effect of host density.

The best models showing the community-level variables associated with mean parasite abundance are shown in Table S3. Past precipitation and current and past host density appeared to be the most important variables (Table 4; Figure 3).

**Table 4:** Parameters of the best models describing the community level factors associated with mean *P. torquans* abundance in a given week.

Term	Coefficient	Standard error	95%CI LB* ^1^	95%CI UB* ^2^
Intercept	−2.001	1.033	−4.026	0.024
**Max. temp. _t−6_**	**0.009**	**0.025**	**−0.040**	**0.058**
**Min. temp. _t−5_**	**0.032**	**0.010**	**0.012**	**0.052**
**Rain _t−4+t−5_**	**0.007**	**0.001**	**0.004**	**0.009**
**Host dens. _t−3_**	**0.815**	**0.140**	**0.541**	**1.089**
**Non passerine dens. _t−1_**	**−1.629**	**0.537**	**−2.682**	**−0.575**
**Non passerine dens. _t−4_**	**−0.815**	**0.327**	**−1.457**	**−0.174**
***Pi. sulphuratus*** ** dens. _t−0_**	**2.528**	**0.299**	**1.943**	**3.114**
***Ph. ruber*** ** dens. _t−2_**	**−0.988**	**0.425**	**−1.821**	**−0.154**
**Predominant host dens. _t−6_**	**1.090**	**0.178**	**0.741**	**1.438**
**Max. temp. _t−6_** * **Year** (**II**)	**0.081**	**0.037**	**0.009**	**0.154**
**Non passerine dens. _t−1_** * **Site** (**reserve**)	**2.122**	**0.615**	**0.917**	**3.328**
**Non passerine dens. _t−4_** * **Site** (**reserve**)	**0.868**	**0.351**	**0.180**	**1.555**
Site (reserve)^a^	0.632	0.337	−0.029	1.293
Year (II)^b^	−2.692	1.073	−4.796	−0.588

Terms in bold indicate the variables of interest. Significant coefficients are underlined.

* ^1^: lower bound;

* ^2^: upper bound.

a : Reference site: Mihura.

b : Reference year: I (2008–2009).

Max.temp: weekly mean maximum temperature; Min.temp: weekly mean minimum temperature; Rain: total precipitation (mm) fallen during a given week. Host dens.: density of nestlings that are potential hosts for *P. torquans*; Non passerine dens.: densities of non passerine birds. *Pi. sulphuratus* dens. and *Ph. ruber* dens.: densities of *Pitangus sulphuratus* and *Phacellodomus ruber* nestlings, respectively; Predominant host dens.: densities of *Pi. sulphuratus*+ *Ph. ruber* + *Ph. sibilatrix* nestling. Year (II): season 2008–2010.

There was a strong positive association between mean larval abundance and the precipitation 4 and 5 weeks previously. The mean minimum temperature 5 weeks in the past was also positively associated with mean *Philornis* abundance. Past maximum temperature (6 weeks previously) showed a weak, though significant, positive association with mean parasite abundance during the season 2009–2010, but such an association was not observed during the first breeding season.

The density of suitable hosts was also a very important driver of *Philornis* abundance (Table 4; Figure 3). The most strongly positively associated variables were current *Pi. sulphuratus* density, past predominant host density (6 weeks previously) and past potential host species (3 weeks previously). Increases in such densities resulted in strong increases in mean parasite abundance at the community level. On the contrary, very significant negative associations were observed with past densities of non-passerine birds in one of the study sites. In Mihura, increases in the density of non-passerine nestlings in that site were followed by decreases in the mean larval abundance in the community, but this association was not present in the reserve. Less strong but consistently significant in both sites was a negative association with *Ph. ruber* two weeks previously.

### Interannual comparisons

The data of the interannual comparisons are shown in Table 1.

**Figure 4:**
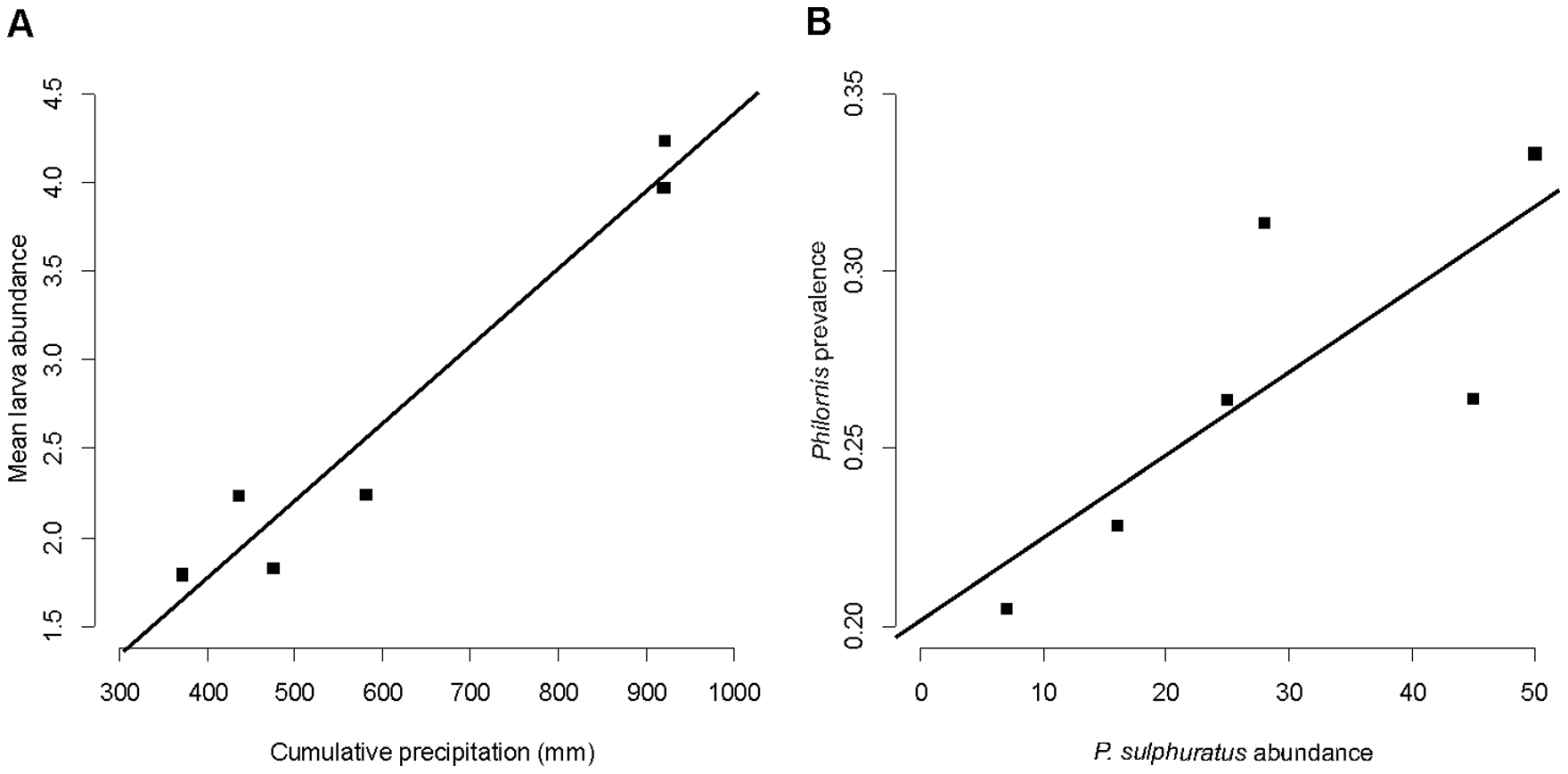
Interannual comparisons with data collected in 6 breeding seasons (2006–2012). Correlation between A) precipitation fallen during the first two thirds of the breeding season and the mean *Philornis* abundance recorded that season; and B) number of *P. sulphuratus* nestlings present in one season and *P. torquans* prevalence in the whole community.

The only statistically significant associations observed in this preliminary analysis were one between cumulative precipitation during the first two thirds of the season and mean parasite abundance (Rho = 0.943; p = 0.017) and between *Pi. sulphuratus* abundance and annual prevalence (Rho  = 0.943; p = 0.017) (Figure 4).

## Discussion

Host-parasite interactions may be influenced by mechanisms operating at a range of biological levels. Studies at different scales of hierarchy contribute to a better understanding of the ecology of wildlife diseases [24]. Such an approach allows a more holistic perspective, thus unveiling the relative size of the effects and facilitating the elucidation of the mechanisms underlying the dynamics of parasites. However, because of its difficulty, such an integrative approach has seldom been used. Although there are prior attempts [25], it was not until the late 1990's that multi-level (or hierarchical) disease studies were seriously undertaken (reviewed by Diez Roux [26]). These hierarchical approaches mostly consisted of including variables from different hierarchical scales in the same statistical models. While this is an appropriate procedure to control for the potential confounding effect of higher-level variables, the inference about the effect of higher level variables may be flawed [27], [28]. We used a stratified analytical approach using different units of analysis, as it can yield more informative and robust results. We evaluated the factors determining the variation in *P. torquans* abundance using four different study units at increasing hierarchical scales, from the intimacy of an individual host, to the dynamic complexity of the bird community. Changing the level of the study unit allows us to truly establish how interactions at lower levels of the biological hierarchy might affect the parasitism dynamics at higher levels. Moreover, examining the effects across the hierarchical gradient provides the opportunity to assess the true biological significance of an observed association. For example, as *P. torquans* frequently parasitizes *Ph. ruber* one may expect that the dynamics of this parasite are positively influenced by the abundance of broods of this bird species. However, the hierarchical model with the individual as the study unit shows a very strong negative influence of *Ph. ruber* density on the number of larvae per nestling (Table 2). When following the community in time, however, this negative association between *Ph. ruber* density and *Philornis* abundance persists, but at a much lower magnitude (Table 4; Fig. 3; e.g. a 10-fold increase in previous *Ph. ruber* density results in a 200-fold decrease in current larval abundance per bird in the individual level model, but only a 50% decrease in the community level model). Yet at a longer time scale, the influence of *Ph. ruber* density was not strong enough to be detected. This clearly shows that using a single hierarchical model with the individual as the study unit would overestimate the negative influence of *Ph. ruber* density on *Philornis* dynamics.

We found several significant associations at all levels, but only few variables truly governed the dynamics of this parasite. At the individual level, the infection was determined by the species and the age of the host. The main driver of parasite abundance at the microhabitat level was the average height of the forest, and at the community level, the density of hosts and prior rainfall.

### Individual level

The abundance of *P. torquans* larvae was largely influenced by host species. Even though a previous study in the same region showed that three species were parasitized above the rest in prevalence and intensity [7], here we find a very marked difference between these predominant hosts, with *Pi. sulphuratus* the most frequently and intensively parasitized. However, this host preference was highly dependent on the density of *Pi. sulphuratus* two weeks previously. Adult flies seek the preferred host, *Pi. sulphuratus*, but when its broods are scarce they can adapt to other hosts, principally *Phacellodomus* in the region studied. This host-preference plasticity determines a temporary host-straggling process that allows the parasite to persist when the main host is not present. However, the 2-week delay is puzzling. It is noteworthy that this 2-week lag was consistently found at different scales of analysis in both negative and positive associations with densities of different host species. It might be related to the behavior of adult flies, which is largely unknown. One possible explanation is that after emerging from the puparium, the flies seek an active nest to develop their adult stage, and it may take some days until gravid females are ready to lay their larvae on the nestlings.

Nestlings are susceptible to parasitism by *Philornis* for the whole duration of the nestling period, but previous reports showed that the number of larvae increases with age, reaching a peak by the end of the second third of the period, after which it declines [10], [29], [30]. The explanation offered for this pattern is that body surface available, the tolerance to parasitism and chances of being found by an adult fly increases as the chick ages, but older nestlings will fledge before the larva can complete development and therefore the parasite would risk not finding a suitable substrate for pupating [10], [30]. In our study, the relationship between nestling age and larval abundance was consistent with this pattern, but the peak took place at very different ages depending on the host species. These different temporal patterns of infection are particularly striking considering that it is the same parasite species, and suggests there might be a plasticity in the parasite synchronization [31], or perhaps a differential immunocompetence, immune response, or a dissimilar maturation of the immune system between species [32]. Further studies should be aimed at elucidating the mechanisms behind this pattern.

### Microhabitat level

The influence of vegetation structure surrounding the nest on *Philornis* abundance was remarkable. We found significant associations with every vegetation stratum. While nest height had no effect on brood infection, the consistent and strong general pattern was a reduction in mean larval abundance as the average forest height increased (except in forests dominated by the only exotic species). This suggests that what is important for the parasite is not the height at which its hosts are, but the microenvironment associated with differential forest height. The presence of medium stratum and the lower stratum height were also associated with *P. torquans* abundance. Segura and Reboreda [8], studying *Pa. coronata* broods, did not find an association between *P. seguyi* and vegetation structure. On the other hand, Le Gros et al. [33] did find an association with vegetation structure, but contrary to our findings, as they observed a greater proportion of nests parasitized by *Philornis porteri* in grasslands than in bushlands or forests, indicating that the influence of the vegetation structure on the dynamics of *Philornis* parasitism depends on the parasite species involved. This strong association with vegetation structure alerts us to the impact that land use change, deforestation and other disruption of the forests caused by humans may have on the dynamics of this host-parasite interaction.

Regarding the more intimate microhabitat, the nest factors studied were not found to be important (only associations explained by host species were found). Species that build the nest with similar materials and structure (*Ph. sibilatrix* and *Ph. ruber*) showed significant differences in their levels of *Philornis* parasitism. Likewise, *S. flaveola*, which uses abandoned *F. rufus* nests to breed, had much less larval abundance than the latter, indicating that the species is more important than the nest characteristics.

### Community level and inter-annual comparisons

Our finding that climatic variables, namely precipitation and temperature, were positively associated with mean larval abundance coincide with previous myiasis studies [6], [7], [34]. The time lags involved might provide clues on the duration of parts of the life cycle of the fly, which is so far ignored. The association with precipitation was much stronger than the one with temperature, and was clearly evident in the preliminary inter-annual comparison.

Other strong driver of *Philornis* abundance at the community level was host density. High densities of *Pi. sulphuratus* nestlings resulted in concomitant high mean larval abundance in the community, which merely indicates that the main host is particularly abundant during that week and so the chances of finding larvae are high, as when we re-ran the analysis excluding *Pi. sulphuratus* hosts in the response (i.e. mean larval abundance in hosts other than *Pi. sulphuratus*) such an association disappeared, showing that more *Pi. sulphuratus* did not result in higher parasitism in other hosts (data not shown). The positive association with past densities of predominant hosts was expected, as many suitable hosts in the past meant more resources for larva to proliferate resulting in greater production of adult flies. This delayed-density dependence of parasite abundance has been documented for several host-parasite systems [35]. Significant negative influences of past host abundances were inconsistent. In one of the study sites, at identical densities of predominant hosts, we observed that high past densities of non-hosts were associated with a reduction in the mean larval abundance in the community, suggesting a dilution effect [23]. The dilution effect has been demonstrated for a tick-borne disease [36] and a rodent virus transmitted by direct contact [37], but never for a myiasis. However, our results should be interpreted with caution, as the association was found in only one site, and when we re-ran the analysis at the community level focusing on the main host (i.e. the response variable was larval abundance on *Pi. sulphuratus*), the association with past non-passerine nestling density disappeared.

Densities of *Ph. ruber* also showed a negative association with *Philornis.* High densities were followed by a reduction in *Philornis* abundance two weeks later. A hypothesis that would explain this unexpected result is that the parents and older *Ph. ruber* nestlings may feed on adult *Philornis* flies that approach to lay their propagules or as they hatch from the pupae that are lodged in their nests (*Ph. ruber* is an insectivorous species).

Time lags appeared to very important in this system, especially for the community-level analysis. It should be taken into account that using many time lags may present over-fitting problems [38]. However, the strength of the associations found in our models argues against the probability of incurring in a type 1 error due to over-fitting.

The preliminary interannual comparison showed that *Philornis* abundance was associated with the precipitation during the first two thirds of the breeding season, and that the annual prevalence depended on the number of *Pi. sulphuratus* nestlings present in the area. It is remarkable to observe that with such limited statistical power (n = 6) we found two statistically significant associations, which indicates that the relationships are of great magnitude, consistent with what was observed at lower time and hierarchy scales. Moreover, an exploratory multivariable linear model found three variables significantly positively associated with mean parasite abundance: precipitation, *Pi. sulphuratus* abundance, and mean minimum temperature (adjusted R^2^ = 0.9996; see Table S4).

Dudaniec et al. [39] observed *P. downsi* parasitizing nestlings at higher intensities in years of high precipitation. They interpreted that increases of *P. downsi* intensity in rainy years were determined by a positive response of host numbers to rainfall, and not by a direct effect of rainfall or temperature. Here we show that the combination of multi-level and multi-variable analyses allows us to dissect the effects of host density from those of climatic variables. Besides the significant influence of host density, we found an additive direct effect of temperature and rainfall.

It is noteworthy that both climatic and host density variables were consistently found to be important predictors in the lower hierarchy models.

## Conclusions

The stratified multi-level approach employed permitted an integrative perspective, unveiling the relative size of the effects and the assessment of interactions between variables at different levels, discriminating between critical and less important factors, and enabling us to observe that the direction and strength of the main relationships were consistent across hierarchical levels and in some cases even through different time scales. For instance, rainy weeks were followed by weeks of increased parasite abundance, and rainy years yielded greater overall parasite production. This helps us realize that precipitation is a driver of *Philornis* abundance both in the short and the long term. Other major driver that consistently appeared strongly associated with *Philornis* infection across all hierarchy scales was the density of suitable host nestlings. These two findings entail that there is potential for global environmental change to affect the impact of *Philornis* on their hosts. Forest fragmentation is resulting in high brood densities in the remaining forest patches [40], and in some areas of South America the weather is becoming wetter and warmer [7], [41], a scenario that might enhance conditions for *Philornis* (although flies are likely to do poorly at higher temperatures not measured in our study).

A broader perspective proved to be more informative than a focalized approach. For instance, the vast majority of *Philornis* studies placed the focus on a single host species. In this study we demonstrated that the infection pattern of the same parasite species along the duration of the nestling period was largely different in each of the three host species examined. At the same time, studying specific relationships at lower levels of the biological hierarchy produces complementary information that helps unveil underlying mechanisms. For example, an investigation of the immune response against *Philornis* in *Phacellodomus* spp. and *Pi. sulphuratus* would establish if there are differences in tolerance and resistance between these host species, which would in turn contribute to understanding why there is such a marked host preference, and why months and years with high *Pi. sulphuratus* abundance result in months and years with greater *P. torquans* abundance, respectively.

A stratified multi-level analysis proved useful to improve our mechanistic understanding of ecological processes associated with parasites and to assess at higher levels the net effect of processes interacting at lower levels. Biogeographical studies and long-term data sets will be crucial for characterizing the context under which these net effects may be different.

Further studies using experimental approaches should explore in more detail the associations identified here to determine its causal nature and establish their underlying mechanisms. In addition, this kind of integrative empirical data may prove invaluable to support theoretical research aimed at explaining and predicting how parasitism at the level of individuals scales up into complex ecological processes [24].

## Supporting Information

Table S1 **Description of the data obtained at different hierarchical levels.**
(DOC)

Table S2 **Terms included in the models at each hierarchical level.**
(DOC)

Table S3 **Selected models that accounted for the 90% of AIC/QAIC cumulative weight for each hierarchical level, showing the main predictors associated with** ***Philornis torquans*** **abundance.**
(DOC)

Table S4 **Linear model with mean** ***Philornis*** **abundance as the response and precipitation, mean minimum temperature and number of** ***Pi. sulphuratus*** **nestlings as the predictors.**
(DOC)

Table S5 **Parameters of the best models describing microhabitat** (**nest and surrounding**) **factors associated with mean** ***P. torquans*** **abundance in a brood.**
(DOC)
