# Supplementary material for: Multi-Level Determinants of Parasitic Fly Infection in Forest Passerines
Source: PLoS One. 2013 Jul 10;8(7):e67104. doi: 10.1371/journal.pone.0067104 (PMC3707910; doi:10.1371/journal.pone.0067104)
Supplement: Table S1 — Description of the data obtained at different hierarchical levels. (DOC) [file pone.0067104.s001.doc]

**Supporting information.**

Table S1: Description of the data obtained at different hierarchical levels.

| Individual-level | | |
| --- | --- | --- |
| *Variable (code)* | *Levels* | *Description* |
| Host species (host sp.) | *Pitangus sulphuratus* | The three predominant host species parasitized by *Philornis torquans* |
|  | *Phacellodomus ruber* |  |
|  | *Phacellodomus sibilatrix* |  |
|  |  |  |
| Age (age – age^2^) | Continuous | Days from hatching (lineal and quadratic terms) |
|  |  |  |
| White blood cells count (WBC) | Continuous | Number of white blood cells/ microlite of blood. (cell/µl) |
|  |  |  |
| Red blood cells count (RBC) | Continuous | Number of red blood cells/microlite of blood. (cell/µl) |
|  |  |  |
| Body mass (bm) | Continuous | Nestling body mass (g) |
|  |  |  |
| Tarsus (tarsus) | Continuous | Tarsus length (mm) |
|  |  |  |
| Score (score) | Continuous | Body mass/ tarsus length (g/mm) |
|  |  |  |
| Other ectoparasite (other ectoparasite) | Presence | Presence or absence the other ectoparasite (eg. mites) |
|  | absence |  |
|  |  |  |
|  |  |  |
| Microhabitat-level | | |
| *Variable (code)* | *Levels* | *Description* |
| Brood species (brood sp.) | *Furnarius rufus* | Brood species parasitized by *Philornis torquans* |
|  | *Paroaria coronata* |  |
|  | *Pitangus sulphuratus* |  |
|  | *Phacellodomus ruber* |  |
|  | *Phacellodomus sibilatrix* |  |
|  | *Sicalis flaveola* |  |
|  |  |  |
| Brood size (brood size) | Discrete | Number of nestling in the nest |
|  |  |  |
| Nest type (nest type) | *Furnarius rufus* | Nest building species |
|  | *Paroaria coronata* |  |
|  | *Pitangus sulphuratus* |  |
|  | *Phacellodomus ruber* |  |
|  | *Phacellodomus sibilatrix* |  |
|  |  |  |
| Parasitic bird (parasitic bird) | Presence | Presence or absence of parasitic bird (*Molothrus bonariensis, Molothrus rufoaxilaris* or *Tapera naevia*) |
|  | Absence |  |
|  |  |  |
| Open/close nest (o/c nest) | Open nest | Nest cover or not by vegetation |
|  | Close nest |  |
|  |  |  |
| External material of the nest (external nest) | Grass | External material of the nest. |
|  | Little sticks |  |
|  | Mud |  |
|  | Nest-box |  |
|  | Sticks |  |
|  | Wood |  |
|  |  |  |
| Internal material of the nest (internal nest) | Fibers | Internal material of the nest. |
|  | Grass |  |
|  | Soft |  |
|  | Sticks |  |
|  | Wood |  |
|  |  |  |
| Height nest (height) | Continuous | Height nest from soil (in meters) |
|  |  |  |
| Support (support) | *Acacia caven* | Tree species that support the nest |
|  | *Aspidosperma quebracho blanco* |  |
|  | *Celtis tala* |  |
|  | *Geoffroea decorticans* |  |
|  | *Gleditsia triachantos* |  |
|  | *Prosopis sp.* |  |
|  | *Sapium haematospermum* |  |
|  |  |  |
| Nestling exposed/don’t exposed | Exposed | Nestlings exposed or don’t exposed to the nest surrounding. |
| (e/ne nestling) | Non exposed |  |
|  |  |  |
| Presence/absence of water (water) | Presence | Presence or absence of an area of water (puddle, river, lagoon, etc) |
|  | Absence |  |
|  |  |  |
| Forest type (tree sp) | *Acacia caven* | Predominant tree species where is the nest (higher stratum) |
|  | *Aspidosperma quebracho blanco* |  |
|  | *Celtis tala* |  |
|  | *Geoffroea decorticans* |  |
|  | *Gleditsia triachantos* |  |
|  | *Prosopis sp.* |  |
|  |  |  |
| Cover by tree (cover tree) | Discrete variable: between 1 (low coverage) to 5 (high coverage) | Degree of forest cover the nest |
|  |  |  |
| Height of the predominant tree (tree height – (tree height)^2^) | Continuous | Mean height of the dominant tree where is the nest, lineal and quadratic term (m – m^2^) |
|  |  |  |
| Presence /absence of bush (p/a bush) | Presence | Presence or absence of medium stratum |
|  | Absence |  |
|  |  |  |
| Soil cover by bush (bush cover) | Discrete variable: from 1 (low coverage) to 5 (high coverage) | Degree of soil cover by shrubs |
|  |  |  |
| Mean height of the predominant bush (bush height – (bush height)^2^) | Continuous | Mean height of the medium stratum where is the nest, lineal and quadratic term (m – m^2^) |
|  |  |  |
| Presence /absence of lower stratum (p/a grass) | presence | Presence or absence of lower stratum |
|  | absence |  |
|  |  |  |
| Soil cover by lower stratum (grass cover) | Discrete variable: from 1 (low coverage) to 5 (high coverage) | Degree of soil cover by lower stratum |
|  |  |  |
| Mean height of the lower stratum (grass height – (grass height)^2^) | Continuous | Mean height of the lower stratum where is the nest, lineal and quadratic term (m – m^2^) |
| Community-level | | |
| *Variable (code)* | *Levels* | *Description* |
| Site (site) | Reserva | Studies sites |
|  | Mihura |  |
|  |  |  |
| Year (year) | I | Studies year. The first year corresponds to reproductive season “2008-2009” and year II to “2009-2010” |
|  | II |  |
| Week (week – week^2^) | Discrete variable | Groups of 7 days (lineal and quadratic terms) |
|  |  |  |
| Mean temperature (mean.temp) | Continuous | Weekly mean temperature the environment |
|  |  |  |
| Maximum temperature (max.temp) | Continuous | Weekly mean of maximum temperature of the environment |
|  |  |  |
| Minimum temperature (min.temp) | Continuous | Weekly mean of minimum temperature of the environment |
|  |  |  |
| Mean humidity (mean.hum) | Continuous | Weekly mean humidity of the environment |
|  |  |  |
| Humidity at 2 am (hum2) | Continuous | Weekly mean of humidity at 2 am of the environment |
|  |  |  |
| Humidity at 2 pm (hum14) | Continuous | Weekly mean of humidity at 2 pm of the environment |
|  |  |  |
| Rain (rain) | Continuous | Weekly sum of precipitation |
|  |  |  |
| Host density (host dens) | Continuous | Density of nestlings that are potential hosts for *P. torquans* |
|  |  |  |
| Shannon’s diversity index  (Shannon’s index) | Continuous | Shannon’s diversity index |
|  |  |  |
| Non passerine birds density (non passerine dens.) | Continuous | Density of non-passerine birds. |
|  |  |  |
| Preferential host density (pref dens) | Continuous | Weekly nestling preference host number/area of site. (Weekly sum the preferential hosts species) |
|  |  |  |
| *Phacellodomus ruber* density (*Ph.ruber* dens.) | Continuous | Weekly *Ph. ruber* nestling number/ area of site |
|  |  |  |
| *Phacellodomus sibilatrix* density (*Ph.sibilatrix* dens.) | Continuous | *Ph. sibilatrix* nestling number/ area of site |
|  |  |  |
| *Pitangus sulphuratus* density (*Pi.sulphuratus* dens.) | Continuous | *Pi. sulphuratus* nestling number/ area of site |
|  |  |  |
| L3 (L3) | Discrete variable | Weekly total of instar three larvae |
