# Supplementary material for: Multi-Level Determinants of Parasitic Fly Infection in Forest Passerines
Source: PLoS One. 2013 Jul 10;8(7):e67104. doi: 10.1371/journal.pone.0067104 (PMC3707910; doi:10.1371/journal.pone.0067104)
Supplement: Table S2 — Terms included in the models at each hierarchical level. (DOC) [file pone.0067104.s002.doc]

**Supporting Information.**

Table S2. Terms included in the models at each hierarchical level.

| Individual level | Microhabitat level | Community level |
| --- | --- | --- |
| *Random intercept* | | |
| IDnestling/IDnest | IDnest | - |
| *Response variable* | | |
| *Philornis* abundance (larvae/nestling) | Mean *Philornis* abundance (larvae/brood) | Mean *Philornis* abundance (larvae/10 nestlings at week*_i_*) |
| *Variables of interest* | | |
| host sp | brood sp | max.temp_t0 – t6_ |
| Age | nest type | min.temp_t0 – t6_ |
| age^2^ | o/c nest | hum2_t0 – t6_ |
| WBC_t-1 - t-2_ | external nest | hum14_t0 – t6_ |
| RBC _t-1 - t-2_ | Internal nest | rain _t0 – t6_ |
| bm _t-1 - t-2_ | height |  |
| tarsus_t-1 - t-2_ | support | *Ph. ruber* dens _t0 t-6_ |
| score _t-1 - t-2_ | e/ne nestling | *Pn. sibilatrix* dens _t0 t-6_ |
| other ectoparasite |  | *Pi. sulphuratus* dens _t0 t-6_ |
|  | water | Pref dens _t0 t-6_ |
|  |  | Host dens _t0 t-6_ |
|  | tree sp | Non passerine dens _t0 t-6_ |
|  | cover tree | Shannon’s index _t0 t-6_ |
|  | tree height |  |
|  | (tree height)^2^ | L3 _t0 t-6_ |
|  | p/a shrubs |  |
|  | shrubs cover |  |
|  | shrub height |  |
|  | (shrub height)^2^ |  |
|  | p/a grass |  |
|  | cover grass |  |
|  | grass height |  |
|  | (grass height)^2^ |  |
|  |  |  |
| *Interactions* | | |
| age*RBC | Tree sp*tree height (lineal term) | Site*men.temp _t-1 – t-6_ |
| RBC*bm | Tree sp*tree height^2^(quadratic term) | Site*max.temp _t-1 – t-6_ |
| RBC*score | Tree sp*height of the nest | Site*min.temp _t-1 – t-6_ |
| host sp*bm _t-1;t-2_ | Brood sp*tree sp | Site*mean.hum _t-1 – t-6_ |
| host sp*WBC _t-1;t-2_ | Brood sp*tree height | Site*hum2 _t-1 – t-6_ |
| host sp*RBC _t-1;t-2_ | brood sp*external material | Site*hum14 _t-1 – t-6_ |
| host sp*score _t-1;t-2_ | brood sp*internal material | Site*rain _t-1 – t-4_ |
| host sp*age | Brood sp*bush cover | Site*pref dens _t-1 – t-6_ |
| host sp*age^2^ | Brood sp*p/a bush | Site**Ph.ruber* dens _t-1 – t-6_ |
| host sp*score*age | Brood sp*p/a grass | Site**Ph.sibilatrix* dens _t-1 – t-6_ |
| host sp*brood size | brood sp*height of nest | Site**Pi. sulphuratus* dens _t-1 – t-6_ |
| site* host sp *age |  | Site*L3 _t-1 – t-6_ |
| site* host sp | Site*brood sp | year*men.temp _t-1 – t-6_ |
| site* host sp * bm | Site*tree sp | year *max.temp _t-1 – t-6_ |
| site* host sp * score | Site*tree height | year *min.temp _t-1 – t-6_ |
| site* brood size | Site*tree height^2^ | year *mean.hum _t-1 – t-6_ |
| year* host sp *age | Site*p/a bush | year *hum2 _t-1 – t-6_ |
| year* host sp | Site* bush cover | year *hum14 _t-1 – t-6_ |
| year* host sp * bm | Site*height bush | year *rain _t-1 – t-4_ |
| year* host sp * score | Site*p/a grass | year *pref dens _t-1 – t-6_ |
| year* brood size | Site* grass cover | year **Ph.ruber* dens _t-1 – t-6_ |
| Rain _t-1 – t-4_* host sp * age | Site*height grass | year **Ph.sibilatrix* dens _t-1 – t-6_ |
| Rain _t-1 – t-4_* age | year*brood sp | year **Pi. sulphuratus* dens _t-1 – t-6_ |
| Rain _t-1 – t-4_* host sp | year *tree sp | year *L3 _t-1 – t-6_ |
| Temp min _t-1 – t-6_*host sp * age | year *tree height | rain*mean.temp _t-1 – t-6_ |
| Temp min _t-1 – t-6_* age | year *tree height^2^ | rain*max.temp _t-1 – t-6_ |
| Temp min _t-1 – t-6_*host sp | year *p/a bush | rain*min.temp _t-1 – t-6_ |
| Pref dens _t0 – t-4_*host sp *age | year * bush cover | host dens _t-1 – t-6_*rain _t-1 – t-4_ |
| Pref dens _t0 – t-4_*host sp | year *height bush | host dens _t-1 – t-6_*mean.temp _t-1 – t-6_ |
| Pref dens _t0 – t-4_*age | year *p/a grass | host dens _t-1 – t-6_*max.temp _t-1 – t-6_ |
| *Pi.sulphuratus* dens_t0–t-6_*host.sp*age | year * grass cover | host dens _t-1 – t-6_*min.temp _t-1 – t-6_ |
| *Pi. sulphuratus*  dens _t0 – t-6_* age | year *height grass | pref dens _t-1 – t-6_*rain _t-1 – t-4_ |
| *Pi. sulphuratus* dens _t0 – t-6_*host sp. | Rain _t-1 – t-4_*brood sp | pref dens _t-1 – t-6_*mean.temp _t-1 – t-6_ |
| *Ph. ruber* dens_t0–t-6_*host.sp*age | Rain _t-1 – t-4_*tree sp | pref dens _t-1 – t-6_*max.temp _t-1 – t-6_ |
| *Ph. ruber* dens _t0 – t-6_* age | Rain _t-1 – t-4_*tree height | pref dens _t-1 – t-6_*min.temp _t-1 – t-6_ |
| *Ph. ruber* dens _t0 – t-6_*host sp. | Rain _t-1 – t-4_*tree height^2^ | *Ph. ruber* dens_t-1 – t-6_*rain _t-1 – t-4_ |
| Non passerine dens _t0 – t-6_* age | Rain _t-1 – t-4_*p/a bush | *Ph.ruber* dens _t-1 – t-6_*mean.temp _t-1–t-6_ |
| Non passerine dens _t0 – t-6_*host sp. | Rain _t-1 – t-4_* bush cover | *Ph. ruber* dens _t-1 – t-6_*max.temp _t-1– t-6_ |
| Non passerine dens_t0–t-6_*host.sp*age | Rain _t-1 – t-4_*height bush | *Ph. ruber* dens _t-1 – t-6_*min.temp _t-1 – t-6_ |
| Non passerine dens _t0 – t-6_* age | Rain _t-1 – t-4_*p/a grass | *Ph. sibilatrix* dens_t-1 – t-6_*rain _t-1 – t-4_ |
| Non passerine dens _t0 – t-6_*host sp. | Rain _t-1 – t-4_* grass cover | *Ph.sibilatrix* dens_t-1– t-6_*mean.temp_t-1–t-6_ |
| Shannon’s index _t0 – t-6_* age | Rain _t-1 – t-4_*height grass | *Ph.sibilatrix* dens _t-1–t-6_*max.temp _t-1–t-6_ |
| Shannon’s index _t0 – t-6_*host sp. | Temp min _t-1 – t-6_*brood sp | *Ph.sibilatrix* dens _t-1–t-6_*min.temp _t-1–t-6_ |
| Shannon’s index _t0–t-6_*host.sp*age | Temp min _t-1 – t-6_*tree sp | *Pi.sulphuratus* dens_t-1 – t-6_*rain _t-1 – t-4_ |
| Shannon’s index _t0 – t-6_* age | Temp min _t-1 – t-6_*tree height | *Pi.sulphuratus* dens _t-1 – t-6_*mean. temp _t-1– t-6_ |
| Shannon’s index _t0 – t-6_*host sp. | Temp min _t-1 – t-6_*tree height^2^ | *Pi.sulphuratus* dens _t-1 – t-6_*max. temp _t-1 – t-6_ |
|  | Temp min _t-1 – t-6_*p/a bush | *Pi.sulphuratus* dens _t-1 – t-6_*min. temp _t-1 – t-6_ |
|  | Temp min _t-1 – t-6_* bush cover |  |
|  | Temp min _t-1 – t-6_*height bush | Non passerine dens _t-1 – t-6_*rain _t-1 – t-4_ |
|  | Temp min _t-1 – t-6_ *p/a grass | Non passerine dens _t-1 – t-6_*mean. temp _t-1– t-6_ |
|  | Temp min _t-1 – t-6_* grass cover | Non passerine dens _t-1 – t-6_*max. temp _t-1 – t-6_ |
|  | Temp min _t-1 – t-6_*height grass | Non passerine dens _t-1 – t-6_*min. temp _t-1 – t-6_ |
|  |  | Non passerine dens _t-1 – t-6_*site |
|  | Pref dens _t-0 – t-4_* brood sp | Non passerine dens _t-1 – t-6_*year |
|  | Pref dens _t-0 – t-4_*tree sp |  |
|  | Pref dens _t-0 – t-4_*tree height | Shannon’s index_t-1 – t-6_*rain _t-1 – t-4_ |
|  | Pref dens _t-0 – t-4_*tree height^2^ | Shannon’s index _t-1 – t-6_*mean. temp _t-1– t-6_ |
|  | Pref dens _t-0 – t-4_*p/a bush | Shannon’s index _t-1 – t-6_*max. temp _t-1 – t-6_ |
|  | Pref dens _t-0 – t-4_* bush cover | Shannon’s index _t-1 – t-6_*min. temp _t-1 – t-6_ |
|  | Pref dens _t-0 – t-4_*height bush | Shannon’s index_t-1 – t-6_*site |
|  | Pref dens _t-0 – t-4_ *p/a grass | Shannon’s index _t-1 – t-6_*year |
|  | Pref dens _t-0 – t-4_* grass cover |  |
|  | Pref dens _t-0 – t-4_*height grass |  |
|  | *Pi. sulphuratus* dens _t-0 – t-6_ * brood sp |  |
|  | *Pi. sulphuratus* dens _t-0 – t-6_*tree sp |  |
|  | *Pi. sulphuratus* dens _t-0 – t-6_*tree height |  |
|  | *Pi. sulphuratus* dens _t-0 – t-6_*tree height^2^ |  |
|  | *Pi. sulphuratus* dens _t-0 – t-6_*p/a bush |  |
|  | *Pi. sulphuratus* dens _t-0 – t-6_* bush cover |  |
|  | *Pi. sulphuratus* dens _t-0 – t-6_*height bush |  |
|  | *Pi. sulphuratus* dens _t-0 – t-6_ *p/a grass |  |
|  | *Pi. sulphuratus* dens _t-0 – t-6_* grass cover |  |
|  | *Pi. sulphuratus* dens _t-0 – t-6_*height grass |  |
|  |  |  |
|  | Non passerine dens _t-0 – t-6_*tree height^2^ |  |
|  | Non passerine dens _t-0 – t-6_*p/a bush |  |
|  | Non passerine dens _t-0 – t-6_* bush cover |  |
|  | Non passerine dens _t-0 – t-6_*height bush |  |
|  | Non passerine dens _t-0 – t-6_ *p/a grass |  |
|  | Non passerine dens _t-0 – t-6_* grass cover |  |
|  | Non passerine dens _t-0 – t-6_*height grass |  |
|  |  |  |
|  | Shannon’s index _t-0 – t-6_*tree height^2^ |  |
|  | Shannon’s index _t-0 – t-6_*p/a bush |  |
|  | Shannon’s index _t-0 – t-6_* bush cover |  |
|  | Shannon’s index _t-0 – t-6_*height bush |  |
|  | Shannon’s index _t-0 – t-6_ *p/a grass |  |
|  | Shannon’s index _t-0 – t-6_* grass cover |  |
|  | Shannon’s index _t-0 – t-6_*height grass |  |
|  |  |  |
|  |  |  |
| *Potential confounders* | | |
| mean.tem_t0 t-4_ | mean.tem_t0 t-4_ |  |
| min.tem_t0 t-4_ | mean.tem_t0 t-4_ |
| max.tem_t0 t-4_ | mean.tem_t0 t-4_ |
| hum2 _t0 t-4_ | mean.tem_t0 t-4_ |
| hum14 _t0 t-4_ | mean.tem_t0 t-4_ |
| rain _t0 t-4_ | min.tem_t0 t-4_ |
|  | max.tem_t0 t-4_ |
| *Ph. ruber* dens _t0 t-6_ | hum2 _t0 t-4_ |
| *Ph. sibilatrix* dens_t0 t-6_ | hum14_t0 t-4_ |
| *Pi. sulphuratus* dens_t0 t-6_ | rain _t0 t-4_ |
| Pref dens _t0 t-6_ |  |
| Host den _t0 t-6_ | *Ph. ruber* dens _t0 t-6_ |
| Non passerine dens_t0; t-6_ | *Ph. sibilatrix* dens _t0 t-6_ |
| Shannon’s index | *Pi. sulphuratus* dens _t0 t-6_ |
|  | pref dens _t0 t-6_ |
| Week and week^2^ | Host dens _t0 t-6_ |
|  | Non passerine dens_t0; t-6_ |
| Height nest | Shannon’s index |
| External material of nest |  |
| o/c nest | Week and week^2^ |
|  |  |
| Brood size | Brood size |
|  | Presence/absence of parasite bird |
|  |  |  |
| Site | Site | Site |
| Year | Year | Year |
