# Supplementary material for: Multi-Level Determinants of Parasitic Fly Infection in Forest Passerines
Source: PLoS One. 2013 Jul 10;8(7):e67104. doi: 10.1371/journal.pone.0067104 (PMC3707910; doi:10.1371/journal.pone.0067104)
Supplement: Table S3 — Selected models that accounted for the 90% of AIC/QAIC cumulative weight for each hierarchical level, showing the main predictors associated with Philornis torquans abundance. (DOC) [file pone.0067104.s003.doc]

**Supporting Information**

**Table S3. Selected models that accounted for the 90% of AIC/QAIC cumulative weight for each hierarchical level, showing the main predictors associated with *Philornis torquans*** abundance

| Microhabitat level | | | |
| --- | --- | --- | --- |
| Models | QAIC | ΔQAIC/2 | QAIC weight |
| brood sp + tree sp*(tree height)^2^ + tree height + grass height + p/a grass + min.temp_t-5_ + *Pi. sulphuratus* dens_t0_ + *Pi. sulphuratus* dens_t-5+t-6_ | 3357.17 | --- | 0.41 |
| brood sp + tree sp*tree height + p/a bush + grass height + p/a grass + rain_t-4_ + min.temp_t-5_ + hum2_t-1_ + hum14_t-1_+ *Pi. sulphuratus* dens_t0_ + *Ph.ruber* dens + *Pi. sulphuratus* dens_t5+t6_ | 3359.14 | 0.37 | 0.15 |
| brood sp + tree sp*tree height + p/a bush + grass height + p/a grass + min.temp_t-5_ +*Pi. sulphuratus* dens_t0_ + *Ph. ruber* dens_t0_ + *Pi. sulphuratus* dens_t-5+t-6_ | 3359.14 | 0.37 | 0.15 |
| brood sp + tree sp*(tree height)^2^ + tree height + parasitic bird + grass height + p/a grass + min.temp_t-5_ + *Pi. sulphuratus* dens_t0_ + *Pi. sulphuratus* dens_t5+t6_ | 3359.17 | 0.37 | 0.15 |
| brood sp + o/c nest + tree sp + tree sp*tree height + p/a bush + grass height + p/a grass + rain_t-4_ + min.temp_t-5_ + hum2_t-1_ + hum14_t-1_+ *Pi. sulphuratus* dens_t0_ + *Ph. ruber* dens_t0_ + *Ph. sibilatrix* dens_t-2_ + *Pi. sulphuratus* dens_t-5+t-6_ | 3360.95 | 0.15 | 0.06 |
| Community level | | | |
| Models | AIC | ΔAIC/2 | AIC weight |
| Rain_t-4+t-5_ + max.temp _t-6_ * year + host dens_t-3_ + *Pi. sulphuratus* dens + *Ph. ruber* dens_t-2_ + predominant host dens_t-6_ + site*Non passerine dens._t-1_ | 417.4 | --- | 0.60 |
| Rain_t-4+t-5_ +min.temp _t-5_  + host dens_t-3_ + *Pi. sulphuratus* dens + *Ph. ruber* dens_t-2_ + predominant host dens_t-6_ + site * Non passerine dens_t-4_ | 418.92 | 0.467 | 0.28 |

References: brood sp: brood species parasitized by *P. torquans*; o/c nest: nest surrounded or not by vegetation; tree sp: dominant tree species in the community; thee height: height mean of dominant tree; p/a bush: presence or absence medium stratum; p/a grass: presence or absence lower stratum; grass height: mean height of the lower stratum; parasitic bird: presence or absence of parasitic birds; rain: weekly sum of precipitation; max.temp: weekly mean maximum temperature and min.temp: weekly mean minimum temperature; *Ph. ruber* dens, *Ph. sibilatrix* dens & *Pi. sulphuratus* dens: *Phacellodomus ruber, Phacellodomus sibilatrix* and *Pitangus sulphuratus* nestling density, respectively; predominant host dens: densities of *Pi. sulphuratus* + *Ph. ruber* + *Ph. sibilatrix*; host den: density of nestlings that are potential hosts for *P. torquans*; Non passerine dens.: density of non passerine nestlings; hum2: mean percentage humidity at 2 am; hum14: mean percentage humidity at 2 pm; site: studies site; _t0 – t-6_ refer to time lags (0= current week; 6= six weeks previously).
