# Supplementary material for: Multi-Level Determinants of Parasitic Fly Infection in Forest Passerines
Source: PLoS One. 2013 Jul 10;8(7):e67104. doi: 10.1371/journal.pone.0067104 (PMC3707910; doi:10.1371/journal.pone.0067104)
Supplement: Table S4 — Linear model with mean Philornis abundance as the response and precipitation, mean minimum temperature and number of Pi. sulphuratus nestlings as the predictors. (DOC) [file pone.0067104.s004.doc]

**Supporting Information**

**Table S4. Linear model with mean *Philornis* abundance as the response and precipitation, mean minimum temperature and number of *Pi. sulphuratus*** nestlings as the predictors.

| Model: mean larvae abundance ~precipitation+ *Pi.sulphuratus* abundance+ minimum temperature | | | |
| --- | --- | --- | --- |
| **Term** | **Coefficients** | **Standard error** | **p-value** |
| Intercept | -2.463 | 4.168e-01 | 0.027456 |
| Precipitation | 3.618e-3 | 5.353e-05 | 0.000219 |
| *Pi.sulphuratus* abundance | 1.081e-2 | 8.354e-04 | 0.005915 |
| Minimum temperature | 1.360e-1 | 2.154e-02 | 0.024177 |

Multiple R-squared: 0.9996, Adjusted R-squared: 0.9991
