# Supplementary material for: Multi-Level Determinants of Parasitic Fly Infection in Forest Passerines
Source: PLoS One. 2013 Jul 10;8(7):e67104. doi: 10.1371/journal.pone.0067104 (PMC3707910; doi:10.1371/journal.pone.0067104)
Supplement: Table S5 — Parameters of the best models describing microhabitat (nest and surrounding) factors associated with mean P. torquans abundance in a brood. (DOC) [file pone.0067104.s005.doc]

**Supporting information.**

**Table S5: Parameters of the best models describing microhabitat (nest and surrounding) factors associated with mean *P. torquans*** abundance in a brood.

| Term | Coefficient | Standard error | 95%CI LB*^1^ | 95%CI UB*^2^ |
| --- | --- | --- | --- | --- |
| Intercept | -8.128 | 3.667 | -15.315 | -0.940 |
| **Brood sp (*F. rufus*)**^a^ | **-0.179** | **0.415** | **-0.991** | **0.634** |
| **Brood sp (*Pa. coronata*)**^a^ | **-2.758** | **0.373** | **-3.488** | **-2.028** |
| **Brood sp (*Ph. ruber*)**^a^ | **-0.304** | **0.376** | **-1.041** | **0.433** |
| **Brood sp (*Ph. sibilatrix*)**^a^ | **-1.878** | **0.340** | **-2.544** | **-1.213** |
| **Brood sp (*S. flaveola*)**^a^ | **-3.466** | **0.343** | **-4.139** | **-2.793** |
| **O/c nest (open)**^b^ | **0.025** | **0.026** | **-0.026** | **0.075** |
| **Tree sp (*P. aff. alba*)**^c^ | **8.017** | **3.307** | **1.536** | **14.498** |
| **Tree sp (*A. caven*)**^c^ | **8.744** | **4.627** | **-0.325** | **17.813** |
| **Tree sp (*G. decorticans*)**^c^ | **8.109** | **3.215** | **1.807** | **14.411** |
| **Tree sp (*A. quebracho-blanco*)**^c^ | **7.168** | **4.209** | **-1.082** | **15.418** |
| **Tree sp (*C. tala*)**^c^ | **13.826** | **55.645** | **-95.240** | **122.891** |
| **Tree height** | **0.773** | **0.454** | **-0.118** | **1.664** |
| **Tree height^2^** | **0.003** | **0.021** | **-0.039** | **0.045** |
| **Tree sp (*P. aff. alba*)*tree height** | **-0.552** | **0.201** | **-0.945** | **-0.159** |
| **Tree sp (*A. caven*)*tree height** | **-0.456** | **0.458** | **-1.353** | **0.441** |
| **Tree sp (*G. decorticans*)*tree height** | **-0.438** | **0.192** | **-0.813** | **-0.062** |
| **Tree sp (*A. quebracho-blanco*)*tree height** | **-0.372** | **0.312** | **-0.984** | **0.240** |
| **Tree sp (*C. tala*)*tree height** | **0.250** | **0.753** | **-1.227** | **1.726** |
| **Tree sp (*P.* *aff.* *alba*)*(tree height)^2^** | **-0.060** | **0.018** | **-0.094** | **-0.025** |
| **Tree sp (*A. caven*)*(tree height)^2^** | **-0.088** | **0.070** | **-0.224** | **0.049** |
| **Tree sp (*G. decorticans*)*(tree height)^2^** | **-0.047** | **0.017** | **-0.079** | **-0.014** |
| **Tree sp (*A. qubracho-blanco*)*(tree height)^2^** | **-0.049** | **0.035** | **-0.117** | **0.019** |
| **Tree sp (*C. tala*)*(tree height)^2^** | **-0.243** | **0.773** | **-1.757** | **1.272** |
| **p/a bush (presence)**^d^ | **0.2053** | **0.102** | **0.006** | **0.404** |
| **p/a grass (presence)**^e^ | **0.503** | **0.356** | **-0.195** | **1.200** |
| **Grass height** | **-1.606** | **0.551** | **-2.686** | **-0.525** |
| **Parasitic bird (presence)**^f^ | **0.026** | **0.081** | **-0.133** | **0.185** |
| Min.temp _t-5_ | 0.130 | 0.033 | 0.065 | 0.194 |
| Rain _t-4_ | 0.001 | 0.001 | -0.000 | 0.002 |
| Hum2_t-1_ | 0.001 | 0.002 | -0.004 | 0.005 |
| Hum14_t-1_ | 0.002 | 0.002 | -0.003 | 0.006 |
| *Pi. sulphuratus* dens. _t0_ | 2.091 | 0.754 | 0.613 | 3.570 |
| *Ph. ruber* dens. _t0_ | -1.167 | 0.786 | -2.707 | 0.374 |
| *Ph. sibilatrix* dens._t0_ | -0.075 | 0.090 | -0.252 | 0.101 |
| *Pi. sulphuratus* dens _t-5 + t-6_ | 1.113 | 0.487 | 0.160 | 2.067 |

*^1^ Lower bound

*^2^ Upper bound

^a^**:** Compared to *Pi. sulphuratus* species (brood sp reference); ^b^: Compared to nest surrounded with vegetation (reference); ^c^: *Gledittsia triacanthos* (tree sp reference); ^d^: Compared to bush absence (p/a bush reference); ^e^: Grass absence (p/a grass reference); ^f^: compared to parasitic bird absence (reference)

Terms in bold indicate the variables of interest. Significant coefficients are underlined.

Reference: brood sp: Brood species parasitized by *P. torquans*; o/c nest: nest surrounded or not by vegetation; tree sp: dominant tree species in the community; tree height: mean height of dominant tree; p/a bush: presence or absence of medium stratum; p/a grass: presence or absence lower stratum; grass height: mean height of lower stratum; parasitic bird: presence or absence of parasitic bird; rain: weekly sum of precipitation; min.temp: weekly mean minimum temperature; hum2: mean percentage humidity at 2 am; hum14: mean percentage humidity at 2 pm; *Ph. ruber* dens, *Pi. sulphuratus* dens and *Ph. sibilatrix* dens: *Phacellodomus ruber*, *Pitangus sulphuratus* and *Phacellodomus sibilatrix* nestling density, respectively; _t0 – t-6_ refer to time lags (0= current week; 6= six weeks previously)
